# Group Post-Admission Cognitive Therapy for Suicidality vs Individual Supportive Therapy for the prevention of repeat suicide attempts: a randomized controlled trial

**DOI:** 10.1186/s13063-020-04816-y

**Published:** 2020-10-27

**Authors:** Laurent S. Chaïb, Jorge Lopez-Castroman, Mocrane Abbar

**Affiliations:** 1 Department of Adult Psychiatry, University Hospital, Place du Pr. R. Debré, 30029 Nîmes, Cedex 9 France; 2 Epsylon Laboratory (EA 4556) Dynamic of Human Abilities & Health Behaviors, University of Montpellier 3, Montpellier, France; 3 CIBERSAM (Centro de Investigación en Salud Mental), Carlos III Institute of Health, Madrid, Spain; 4 INSERM U1061, University of Montpellier, Neuropsychiatry: Epidemiological and Clinical Research, Montpellier, France; 5 Montpellier University, Montpellier, France

**Keywords:** Repeat suicide attempts, Randomized controlled trial, Psychosocial interventions

## Abstract

**Background:**

Suicide is a serious public health problem. The development and use of effective treatments for people hospitalized for suicide attempts remain a priority. Regarding psychosocial treatment, the evidence for treatments that effectively prevent suicide repetition of suicide attempts is extremely thin. There is some evidence that cognitive behavioural therapy may be effective for reducing suicide behaviour. The primary aim of this study is to compare Group Post-Admission Cognitive Therapy for Suicidality (GPACTS) versus Individual Supportive Therapy (IST) for preventing suicide.

**Methods:**

In total, 240 participants with a high suicide risk score according to a Mini International Neuropsychiatric Interview (MINI) will be randomized to either GPACTS or IST. This is a multicentre, parallel group, randomized (1:1 ratio), two-tailed-superiority trial with endpoint-assessor blinding. Patients meeting inclusion criteria during a screening visit will be enrolled in the study and randomized into two groups: one group will undergo 6 weeks of GPACTS, and the second group will undergo 6 weeks of IST. Following 6 weeks of interventional therapy, patients are followed up for 12 months. Follow-up for both groups is identical and includes the administration of questionnaires at baseline and then within 10 days after the end of therapy sessions and then at 3, 6 and 12 months following the end of GPACTS/IST sessions.

**Discussion:**

To our knowledge, this is the first RCT of its kind to be conducted in France, and so far, there are no studies in the literature on group psychotherapy for the treatment of individuals who have attempted suicide. The outcomes will provide clear guidance for professionals to apply psychological intervention with suicide attempts. The protocol respects ethical principles, and ethical approval was obtained from the local ethics committee. The results will be disseminated through an original research published as original research in peer-reviewed manuscript, through a therapist manual for cognitive therapy, and presentations at research conferences.

**Trial registration:**

ClinicalTrials.gov NCT02664701. Registered on January 27, 2017.

## Administrative information

**Table:** 

**Title {1}**	Group Post-Admission Cognitive Therapy For Suicidality vs Individual Supportive Therapy for the prevention of repeat suicide attempts: a randomized controlled trial.
**Trial registration {2a and 2b}**	ClinicalTrials.gov Identifier NCT02664701, registered on January 27, 2017.
**Protocol version {3}**	The current protocol version (5.0) was approved on 15 October 2018.
**Funding {4}**	This study was supported by a grant from the French Ministry of Health (PHRC 2014, 140241).
**Author details {5a}**	Laurent Chaïb, PsyD, PhD^1,2^ Jorge Lopez-Castroman, MD, PhD^1, 2, 3, 4^ Mocrane Abbar, MD^1^ ^1^Department of Adult Psychiatry, University Hospital, Nîmes, France ^2^Epsylon Laboratory (EA 4556) Dynamic of Human Abilities & Health Behaviors, University of Montpellier 3, Montpellier France ^2^CIBERSAM (Centro de Investigación en Salud Mental), Carlos III Institute of Health, Madrid, Spain ^3^INSERM U1061, University of Montpellier, Neuropsychiatry: Epidemiological and Clinical Research, Montpellier, France ^4^Montpellier University, Montpellier, Franc
**Name and contact information for the trial sponsor {5b}**	**CHU de Nîmes**, Direction de la Recherche et de l’Innovation, Mr. Nicolas Best, Place du Professeur Debré, 30,029 Nîmes Cedex 09 France; Phone: 04.66.68.42.36; Fax: 04.66.68.34.00; email: drc@chu-nimes.fr
**Role of sponsor {5c}**	The sponsor had no role in the design of this study and will not have any role during its execution, analyses, interpretation of the data, or decision to submit results

## Background and rationale {6a}

Suicidal prevention is an international public health priority. Despite the fact that suicide is one of the leading global causes of unnatural deaths and an enormous economic impact estimated in billions of euros per year in developed countries, suicide prevention strategies must be improved in many countries [1]. It is well documented in the literature that suicide risk is highest in the year after people have been discharged from a psychiatric hospital. A review of the literature confirms that patients recently discharged from hospitals have a risk 100 times higher compared with the general population and this risk peaks in the weeks immediately after discharge [2].

Numerous studies have been conducted to identify variables associated with suicide to help clinicians to determine who is at risk to attempt suicide. Attempted suicide is one of the strongest risk factors for completed suicide in adults. A meta-analysis of follow-up mortality studies estimated that individuals who attempted suicide were 38 to 40 times more likely to commit suicide than those who had not attempted suicide [3]. Other data suggest the need to develop early interventions for this population closer to hospitalization. Indeed, suicide attempters are estimated to have a risk of dying from suicide in the first year following their attempt that is more than 66 times the annual risk of suicide in the general population [4].

The treatment of suicidal behaviour (SB) is one of the most difficult challenges faced by clinicians. Pharmacological and psychosocial interventions are commonly proposed to suicide attempters and are usually offered in tandem. The development and use of effective treatments for people hospitalized for suicide attempts remain a priority. Regarding psychosocial treatment, the evidence for treatments that effectively prevent the repetition of suicide attempts is extremely thin [5]. One of the methodological difficulties associated with conducting these studies is that suicide rate is a rare event and a larger sample size is necessary to show statistically significant differences between a therapeutic group and a control group. However, there is some evidence that cognitive behavioural therapy (CBT) may be effective for reducing SB.

Brown et al. proceeded a randomized controlled trial (RCT) to evaluate the efficacy of cognitive therapy for the prevention of repeat suicide attempts [6]. The sample consisted of 120 patients who attempted suicide and who received a psychiatric evaluation within 48 h of the attempt. Patients were randomly assigned to receive either CBT or treatment as usual. The CBT protocol consisted to receive ten individual therapy sessions according to a treatment manual [6]. Follow-up assessments were conducted on all individuals over an 18-month period to determine whether they made another suicide attempt. The results showed that patients who received cognitive therapy were about 50% less likely to make a repeat suicide attempt during the follow-up period than those who did not receive cognitive therapy. Authors concluded that cognitive therapy was an efficacious intervention for preventing suicide attempts.

A meta-analysis by Tarrier et al. confirms that CBT can reduce SB in the short term [7]. CBT does prove effective when compared with minimal treatment and was still effective when studies using control groups involving active psychological treatments were included in the analysis. This result suggests that CBT has a specific effect. Psychotherapy outcomes are generally thought of as consisting of both specific and non-specific effects. Non-specific effects like emotional support, therapeutic attention, empathic listening, implementation of therapeutic optimism, and others are the result of every successful therapeutic relationship. These contrast with specific effects that are directly targeted by other types of therapy. One of the specific effects of CBT that is well documented is problem-solving strategies [8].

A more recent RCT study with 2-year follow-up was conducted by Rudd et al. [9] who compared brief CBT to treatment as usual for the prevention of suicide attempts in military settings. Results show that soldiers in brief CBT were approximately 60% less likely to make a suicide attempt during follow-up than soldiers in treatment as usual.

CBT could be an important contribution to the prevention of suicide. Moreover, treatment is more effective when directly focused on reducing a specific aspect of SB and less so when focused on other symptoms (such as depression or distress) aimed at reducing SB as a secondary effect. In other words, to be effective, specific CBT suicide prevention treatment programs need to be designed, tailored, and implemented to focus on suicidal behaviour [7]. Despite these favorable preliminary results with CBT, the authors highlight the need for randomized controlled trials with sufficient power to detect treatment differences [9]. More controlled studies are required to establish psychotherapeutic techniques that will impact SB and that clinicians can be more confident with.

In order to respond to this need, this study will compare two types of psychosocial treatment: one with specific and non-specific effects (CBT called Group Post-Admission Cognitive Therapy for Suicidality [GPACTS]) and the other (Individual Supportive Therapy [IST]) with only non-specific effects. We choose to study CBT in a group format because the results of evaluative research on psychotherapy have demonstrated that the format of the therapy (individual versus group) does not appear to predict the outcome for several mental disorders [10]. In addition, the group format provides pragmatic advantages, such as more efficient use of human resources dedicated to patient care and subsequent cost savings. Thus, we hypothesize that GPACTS for suicide attempters can offer advantages in comparison with individual procedures, even if they cannot always perfectly fit the specific needs of every patient.

### Objectives and hypothesis {7}

The primary objective of this study is to evaluate the efficacy of a program of 6 sessions of GPACTS (as compared to 6 sessions of IST) designed for preventing repeat suicide attempts at 12 months post-psychotherapy in adults admitted to inpatient care for suicide attempts. The secondary objectives of this study are to assess the efficacy of GPACTS on parameters describing the incidence of suicide and repeat suicide attempts, as well as suicide reattempt-free follow-up time long-term changes in suicidal ideation and long-term changes in psychiatric symptoms (depression, hope for the future, hospitalization). We expect that patients in the IST group will reattempt suicide at an earlier date and a higher frequency as compared to patients enrolled in the GPACTS.

### Trial design {8}

This is a multicentre, parallel group, randomized (1:1) and two-tailed-superiority trial with endpoint-assessor blinding. Patients meeting inclusion criteria during a screening visit administered by a psychiatrist will be enrolled in the study and randomized into two groups:

- One group will undergo 6 weeks of IST (the comparator group) and
- The second group will undergo 6 weeks of CBGT (the experimental group).

Randomization is carried out by a designated person (e.g. a participating psychologist), who is not a follow-up assessor, following baseline assessments. Interventional therapies will take place once per week for 6 weeks in appropriate facilities at the participating centres. The psychologists in charge of interventional therapies will be trained prior to study start in order to homogenize practices between participating centres. The psychologists in charge of group therapy are not the same as those in charge of individual therapy (to help avoid cross-contamination between arms); pre-study training is similarly separated by therapy type (i.e. a participating psychologist is trained in only one type of psychotherapy, which he/she administers during the study).

## Methods, participants, interventions and outcomes

### Study setting {9}

The clinical aspects of this trial will take place within participating academic or private hospitals (urban setting) located in France. Eight centres (seven academics and one private) have agreed to participate. The list of study site could be obtained by contacting the sponsor.

### Eligibility criteria {10}

#### Participant inclusion criteria

- Has given his/her informed and signed a consent
- Must be insured or beneficiary of a health insurance plan
- Is 18 years of age or older
- Speaks fluent French
- Is freely hospitalized (in centres or via emergency services) for the prevention of suicide
- Has a high suicide risk score according to a Mini International Neuropsychiatric Interview (MINI) structured interview (MINI)
- Prior (or recent) suicide attempt within the last month
- Is able to understand the study and capable of giving his/her informed consent
- Is available during the weekly time slots proposed by the investigator

#### Participant exclusion criteria

- Is participating in another interventional study, or has participated in another interventional study within the past 3 months
- Is in an exclusion period determined by a previous study
- Is under judicial protection, or is an adult under guardianship
- Is impossible to correctly inform the patient, or the patient refuses to sign the consent
- Emergency situations preventing proper study conduct
- Diagnosis of schizophrenia or presence of a psychotic disorder evaluated by the psychiatrist with the MINI at initial assessment
- Serious cognitive impairment and medical incapacity to participate according to the medical file or observed during the initial interview by the psychiatrist
- Severe dependence on any substance (including alcohol and cannabis) according to the MINI
- Current psychotherapy

#### Psychologist inclusion criteria

- Holds the title of psychologist (registration with the “*Agence régionale de la santé”*)
- Authorization to practice psychotherapy (registration with the “*Agence régionale de la santé*”)
- Adhering to the Code of Ethics for Psychologists of the French Federation of Psychologists and psychology
- At least 3 years of psychotherapy practice
- Psychologist trained in either CBT or supportive therapy but not both in order to avoid cross-contamination

#### Psychologist exclusion criteria

- Refuses to participate in key study activities, such as standardizing practices, study-related meetings and training

#### Participating centre inclusion criteria

- The target population is present in the centre
- Capacity to recruit patients so as to respect the study timetable
- Psychologists trained for (or willing to undergo training) and available to perform the proposed psychotherapies
- Appropriate facilities for group therapy that will respect patient confidentiality
- Appropriate facilities for individual therapy that will respect patient confidentiality

#### Participating centre exclusion criteria

- Ongoing interventional clinical trials whose recruitment would significantly interfere with recruitment for the present study

### Who will take informed consent {26a}

This is a biomedical research protocol requiring informed consent of participants as specified in the following sub-sections. An informative letter will be presented to the participant and will state the purpose, the objectives and conduct of the study in accordance with current regulations and their rights to refuse to participate in the study or leave the study at any time. Patient consent will be sought and obtained before the entry thereof in the study. A copy of the signed consent will be given to the patient and a copy will be retained by the investigator; a third copy will be retained by the sponsor. The participating psychiatrists are responsible for correctly informing patients and obtaining their informed consent.

### Additional consent provisions for collection and use of participant data and biological specimens {26b}

This is not applicable because no biological data will be collected.

### Interventions

#### Explanation for the choice of comparators {6b}

Ethical questions and the current state of knowledge about the effectiveness of psychosocial treatment for suicidal persons helped guide us to the choice of IST (instead of “usual care”) as a comparator for CBGT. For example, the notion of “usual care” can lead to a large variation in the quality and efficacy of what happens in the comparator arm. In addition, several studies indicate benefits associated with psychosocial interventions (all interventions) when compared with usual care, thus suggesting IST to be a better and more conservative choice. *Individual* Supportive Therapy was chosen instead of *group* supportive therapy because the latter is not suitable for suicidal attempters, given the informal aspect of it and the risk of contagion of thoughts and/or behaviours among participants.

### Interventions description {11a}

Each center will offer both types of therapy (IST and GPACTS), and each psychologist will only administer one type of therapy.

#### Description of Individual Supportive Therapy

In the comparator group, the “intervention” corresponds to six 60–90-min IST sessions administered for 6 weeks (with one session per week). IST does not rely on specific theories or assumptions about the causes of suicide. IST will be focused on the patients’ daily life experiences. The role of the psychologist will be to structure the interview and its duration. However, with respect to specific processes related to the modification of suicidal beliefs, IST is not a specific treatment, and the strategies from cognitive and behavioural approach will not be used in any way.

The psychologist will accompany and support the client in a non-judgmental and empathic environment, to enable the client to speak and think about himself and his life. During the meeting, the psychologist should never make interpretations, cognitive restructuring, solve problems or give advice. In the same vein, the psychologist should never suggest that what the patient says is good or bad, but on the contrary, bring the patient’s speech with equal and neutral interest. Psychologists have been instructed not to apply strategies based on specific therapies such as CBT and in particular not to apply the following strategies: give advice, make suggestions, problem-solving logical analysis, proofs and/or probabilities, alternative thoughts, identification of beliefs, relaxation strategies, exposure strategies, suggestions for adaptation methods, identify and discuss thought errors, psychological interpretations and use of imaging techniques. In summary, all techniques from CBT especially but also issues specific to other approaches must not be applied.

#### Description of cognitive behavioural group therapy

In the experimental group, the “intervention” corresponds to six 90–120-min group cognitive therapy sessions administered for 6 weeks (with one session per week and 6 persons per group). Sessions must start within 8 weeks after the inclusion date for every patient.

The protocol was adapted from individual face-to-face therapy as described by Ghahramanlou-Holloway et al. [11, 12] for preventing repeat suicide attempts and known as Post-Admission Cognitive Therapy (PACT). In reference to this work, we named our program GPACTS.

PACT was also adapted by the authors from a 10-session CBT program developed by Brown et al. to prevent repeat suicide attempts. As pointed by the authors, PACT is based on Beck’s theories of depression [13] and suicide [6] and serves as a foundation for GPACTS.

GPACTS will consist of six sessions with the same overall therapeutic goals identified by Ghahramanlou-Holloway et al. [11]: (1) to reduce suicidal recidivism, (2) to reduce the impact of psychological risk factors such as depression, chronic suicide ideation and hopelessness, (3) to develop problem-solving skills and coping strategies by linking them to the problems that contributed to the suicidal act, (4) to develop the use of existing social support, (5) to improve the use of and the collaboration with mental health professionals and (6) to help the patients in developing a safety plan including coping strategies to preventing relapse.

All GPACTS group session will be structured as follows: (1) welcome and introduction to the agenda of the session, (2) summarize the previous session and correction of exercises performed at home, (3) work on today’s theme (e.g. learning problem-solving techniques), (4) presentation of exercises to do at home and (5) feedback from participants on the meeting and answering questions.

The six sessions are distributed in three modules as follows:

- Module 1 is called “Understanding” and consisting of sessions 1 and 2. Session 1 provides general information about the program and introduction to CBT and provides psychoeducation about the suicidal crisis. Session 2 is focused on collaboratively generating a cognitive and behavioural conceptualization based on a narrative review of the most recent suicide attempt.
- Module 2 is called “Mastering” and consisting of sessions 3, 4 and 5. In session 3, the problem of hopelessness is introduced through the identification of personal reasons for living and the purpose of constructing a hope box containing different elements (such as letters from friends, coping strategies cards, pictures). The objective of a hope box is to have tangible evidence that life is valuable and make the reasons for living concrete. Session 4 is focused on the impact of low emotional regulation and its contribution to the recent suicide attempt. The need to improve the coping strategies is introduced through teaching and practice progressive muscle relaxation and controlled breathing exercises. In session 5, the patients are introduced to the relationship between problem-solving deficit and suicidal crisis. The patients are invited to identify from their cognitive and behavioural conceptualization for their recent suicide attempts and their personal problem-solving style. The classic steps of problem-solving will be reviewed with the patients: (1) identifying related problems and emotions, (2) generating solutions, (3) weighing pros and cons of solutions, (4) choosing the most realistic solution, (5) carrying out the solution and assessing its outcome.
- Module 3 is called “Preventing”, consisting of session 6. This session is focused on relapse prevention and the construction of the safety plan. In order to prevent relapse, patients are asked from the most recent suicide attempt conceptualization to imagine how the different strategies learned during GPACTS could influence positively the crisis unfold. Finally, a safety plan is individually constructed using an adaptation of the model presented by Stanley et al. [14]. In this form, patients are invited to develop a personal and hierarchical list coping strategies to use in future distressing situations.

### Criteria for discontinuing or modifying allocated interventions {11b}

There will be no special criteria for discontinuing or modifying allocated interventions. Patients will inform at the time of inclusion that they will not be able to choose between IST or GPACTS and that they will not be able to change therapy during the course of the study. They will be also informed that they could stop the therapy at any time. In this case, the patient is re-oriented towards routine care, according to patient needs (as evaluated by the medical teams involved). Statistical analyses however will evaluate data according to initial randomized arm, i.e. on an intent-to-treat basis. Follow-up is continued as described in this protocol (i.e. the stopping of the experimental intervention does not result in exclusion from the study).

### Strategies to improve adherence to interventions and monitoring adherence {11c}

To improve the adherence to the intervention, each centre will propose two time slots for group therapy (e.g. Tuesday evenings at 6 pm or Friday afternoon at 2 pm) and that at inclusion the patients are asked if they are interested in the study and available for both time slots in case they are in the group therapy group. Regarding the adherence monitoring, a register of attendance and absences of patients will be completed by psychologist in both interventions (GPACTS and IST).

### Relevant concomitant care permitted or prohibited during the trial {11d}

Concomitant pharmaceutical treatments are decided by the treating psychiatrist; all prescriptions are recorded in the electronic Case Report Form (eCRF). Patients will be asked to maintain a drug consumption calendar throughout the study. They will also be asked to record additional psychotherapy options in the calendar.

### Provisions for post-trial care {30}

Patients are expected to manage their own transportation to and from visits. Patients may be reimbursed for up to 50€ of transportation costs per added visit, pending provision of corresponding receipts. These transportation costs have been provided for in the PHRC-N 2014 budget.

### Outcomes {12}

Following 6 weeks of interventional therapy, patients are followed up for 12 months by a psychiatrist. Follow-up for both groups is identical and includes the administration of questionnaires at baseline and then within 10 days after the end of GPACTS/IST sessions and then at 3, 6 and 12 months following the end of GPACTS/IST sessions. The primary outcome of interest for this study is the duration of time free of suicide reattempts. The null hypothesis for the primary outcomes for this study is that there will be no significant differences in the duration of a suicide reattempt-free follow-up period between the intervention and the control group.

Several secondary outcomes will be evaluated:

- The Columbia Suicide Severity Rating Scale (C-SSRS). This physician-administered scale prospectively measures the severity and intensity of suicidal ideation, the different types of suicidal behaviour and the lethality of suicide attempts [15]
- The Beck Scale for Suicide Ideation (BSSI) is a 21-item, validated, self-report questionnaire that can be used to identify the presence and severity of suicidal ideation [16, 17]
- The Beck Depression Inventory (BDI-II) is a self-assessment scale. Its purpose is to quantify the intensity of depression [18]
- The Beck Hopelessness Scale (BHS) is a validated questionnaire designed to measure an individual’s expectations about the future [19]
- The Mini International Neuropsychiatric Interview – 7 (MINI) is a tool that helps identify the psychopathology of a subject according to the DSM5 [20]
- The Risk-Rescue Rating Scale (RRRS) assesses the lethality of a suicide attempt, defined as the probability of inflicting irreversible damage. The underlying hypothesis is that lethality can be expressed as a ratio of factors influencing risk and rescue [21]
- Demographic forms and other assessments are used to characterize the sample and control for potential confounders.
  These additional scales provide valid appraisals of factors related to suicidal behavior such as:
- Life events: the Social Readjustment Rating Scale (SRRS [22];), the Childhood Experience of Care and Abuse Questionnaire (CECA-Q [23];)
- Personality traits: Spielberger’s State-Trait Anger Expression Inventory (STAXI-2 [24];), Barratt Impulsiveness Scale (BIS11 [25];), State-Trait Anxiety Inventory (STAI [25];)
- Cognitive functioning: the Cognitive Reflection Test (MIT-IQ [26];)
- Severity of alcohol and tobacco dependence: the CAGE Questionnaire [27] and the Fagerström questionnaires [28]

### Participant timeline {13}

**Fig. 1:**
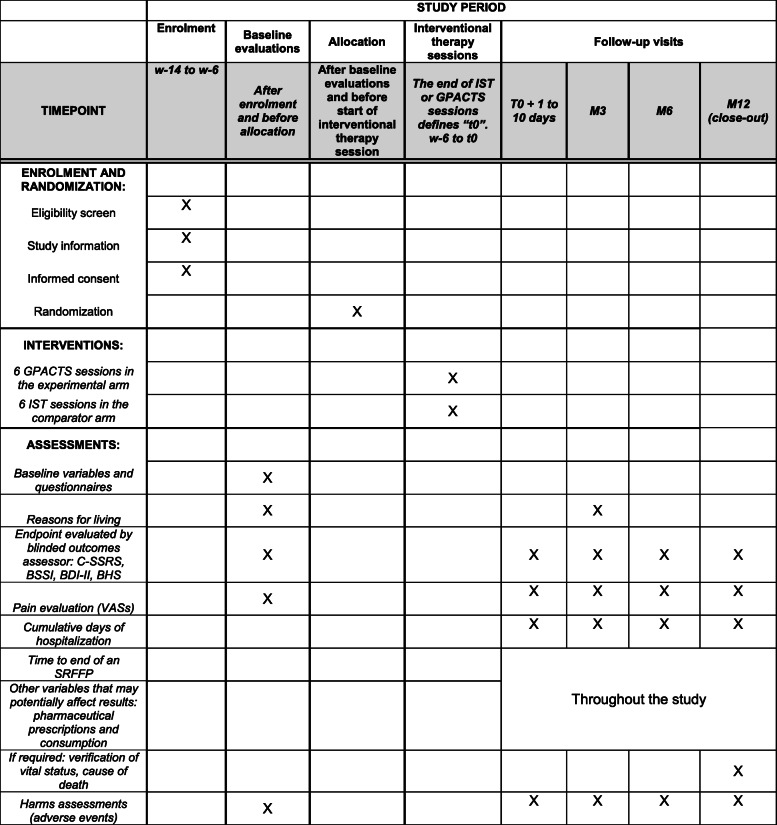
Schedule of enrolment, interventions and assessments according to the Standard Protocol Items: Recommendations for Intervention Trials (SPIRIT) Diagram

The maximum study duration (i.e. starting at enrolment and ending at close-out) for a given patient is 15.5 months. The anticipated study calendar provides for 12 months of inclusion, 15 months of follow-up, 6 months of data management and 6 months of statistical analysis and report writing. The time from inclusion to randomization should not exceed 3 months (see Fig. 1).

### Sample size {14}

We will test the following hypothesis: The mean time to the next suicide attempt during the follow-up period is different between the two groups. The probability of a suicide reattempt-free follow-up period (PSRFFP) according to usual care was estimated at 60% by Brown et al. [6]. In this trial, a cognitive behavioural therapy (10 sessions of individual therapy) was associated with an increase of 20% in the PSRFFP at 12 months.

In our study, the 10 sessions of individual therapy program are replaced by 6 sessions of a group therapy program. We expect a minimum benefit of 20% for this new strategy (60% in the control group versus 80% in the experimental group).

To our knowledge, no study has reported intra-class correlation coefficients associated with therapists or group therapy suicide re-attempt of the C-SSRS score. It is therefore difficult to anticipate an exact sample size that would take into account clustering effects caused by group therapy (or by therapist effects) in the primary outcome assessment.

Under the hypothesis of no cluster effects (at either the therapist or therapy-group levels) and using a log-rank test, 186 subjects are required to detect 20% difference in PSRFFP at 12 months (60% in the control group and 80% in the experimental group), with a power of 85% and a bilateral alpha risk of 5%.

Given the possibility of cluster effects, we have increased this number to 216. Taking into account an anticipated rate of 10% lost to follow-up, 240 patients will be included (120 patients per group).

Patient consulting under emergencies for suicidal behaviour are common. It is more than reasonable to expect at least one case per day per centre on average, giving a minimum potential patient pool of about 1460 patients during the proposed recruitment period. To take into account the time necessary to organize group sessions and the probability that some patients might not want to participate, we proposed an inclusion averaging 5 patients per month per centre.

### Recruitment {15}

Recruitment will be carried out by the participating psychiatrists during visits with potential patients at the eight centres. It is difficult to estimate the available patient pool via hospital statistics because suicide ideation is not coded. However, patients consulting under emergency situations for suicidal behaviour are common (there are over 800 hospitalizations for suicide attempts at the NUH per year). It is more than reasonable to expect at least one case per day per centre on average, giving a minimum potential patient pool of about 1460 patients during the proposed recruitment period. Recruitment of patients started in November 2017 and will be completed in June 2021. The first groups, one that started with GCBT and the other with IST, received treatment from December 2017 to January 2018. In order to take into account the time necessary to organize group sessions and the probability that some patients might not want to participate, we propose an inclusion curve averaging 5 patients per month per centre.

### Assignment of interventions: allocation

#### Sequence generation {16a}

Patients will be randomized to either study arm in a 1:1 ratio. Randomization lists consisting of randomly sized blocks will be established per centre. These lists are the responsibility of the study methodologist at the Department of Biostatistics, Epidemiology, Public Health and Medical Information at the Nîmes University Hospital (BESPIM). A specifically designed SAS program (Cary, NC, USA) will be used to carry out randomization. The number of subjects per block will be known only to the methodologist.

#### Concealment mechanism {16b}

A web application for patient randomization will be created for the needs of the study. Following user login, patient identification (first letter of last name + first letter of first name + year of birth) and verification of screening and exclusion criteria, the study arm will be indicated to the user. The use of a web application thus ensures a high degree of security as concerns randomization: it is impossible to modify the order of randomization; patient assignment to a study arm and a randomization number is definitive. The web application will be implemented by the e-Santé team at the BESPIM.

#### Implementation {16c}

The allocation sequence will be generated by the study methodologist at the BESPIM. Patient enrolment will be carried out by including psychiatrists. Randomization is carried out after patient inclusion and after baseline assessments by participating psychologists (i.e. not the including psychiatrists, who are also the outcome assessors).

### Assignment of interventions: blinding

#### Who will be blinded? {17a}

Baseline assessments will be made before randomization, so these are blinded. Patients cannot be blinded but will be asked to not reveal their group status to anybody outside their group, not even their treating psychiatrist. Furthermore, the hypotheses tested will not be communicated to patients (i.e. the patient will not be informed on the supposed superiority of one group over another).

Therapy care providers (psychologists) cannot be blinded. In order to make assessments as objective as possible, outcome assessors (psychiatrists) will be different from the therapy providers (psychologists), and every attempt will be made to keep outcome assessors blinded to patient group status. To control for the success of blinding, a “guess-the-group” question will be addressed to outcome assessors. Outcome assessor responses will be compared to expected results due to chance. Data analysts will not be involved in trial field logistics and will be blinded. During analyses, when group assignments are first required they will only be revealed as “group A” or “group B”. Only when analyses have been completed will the exact nature of groups be revealed.

#### Procedure for unblinding if necessary {17b}

We do not anticipate any requirement for unblinding. The exact nature of groups will be revealed only when analyses will be completed.

### Data collection and management

#### Plans for assessment and collection of outcomes {18a}

The coordinating psychologist will visit each participating center to present the interventional therapies and homogenize practice between centres. In each participating center, the psychologists will be instructed about one of the interventional therapies (GPACTS or IST) and will receive a treatment manual. This treatment manual will be used during the treatment to help the psychologists to stay focused on interventional therapies.

#### Plans to promote participant retention and complete follow-up {18b}

Upon enrolment and then randomization, patients will be provided with a study calendar and an authorized study technician will help patients organize their follow-up visits. The study technician will regularly telephone participants to remind them of upcoming visits and to perform any necessary rescheduling if possible. Patients will be asked to provide the name and contact information for a trusted person in case of loss of contact with study personnel. In case contact with the patient is lost, study technicians will contact the trusted person or the patient’s generalist in order to re-establish contact and organize study visits. A list of participants lost to follow-up or declared as deceased will be mailed to the Center for Epidemiology on Medical Causes of Death register (CépiDc) to verify the vital status and, if appropriate, the cause of death.

### Data management {19}

Data management will be performed in line with The International Conference on Harmonisation of Technical Requirements for Registration of Pharmaceuticals for Human Use (ICH) requirements. The related documents will be stored on the BESPIM server.

#### eCRF data

eCRF fields are formatted so as to enforce homogenous value types and require confirmation for less probable values and/or out-of-expected-range values: data entries, modifications, who made them and when are fully traceable (complete audit trail). An electronic signature by the investigator engages his/her responsibility for the data in an eCRF.

The eCRF will represent only raw data values as opposed to calculated values, unless the latter are necessary for further eCRF input.

#### eCRF data security

The software used to create eCRFs is hosted on a website within the Nîmes University Hospital (NUH). Access to this software is secured via a password. The data collected through generated eCRFs are subject to daily backup on a secure network. The network is connected to the Internet and the access is protected by a firewall.

Clinical study data will be stored in a specific directory on the server. Only network administrators and authorized persons from the BESPIM may have access to this directory.

The following measures were taken to implement confidentiality:

- The required information technology is located in the BESPIM; access is controlled and secure.
- Data are stored on a server hosted in a secure room at the NUH.

To cope with any hardware or software problem that could occur involving the storage unit of virtual machines, another storage unit that is larger in terms of space, but less swift in terms of throughput, is deployed. Dedicated software (VRangerPro) provides a daily incremental backup of virtual machines (except Saturdays and Sundays). A full backup of these machines is performed every Friday night for an instant return to N-15 days in case of problems.

The hard drives of the virtual servers are installed “en raid”, which ensures IT security in case of hardware failure of one of the disks.

Clinical study data will be stored in a database server. Only network administrators and authorized BESPIM personnel can access this server.

#### eCRF extractions

The extraction of data for analysis will be conducted by authorized BESPIM personnel who possess the necessary rights in the eCRF application.

### Confidentiality {27}

The closing of the trial including the closure of the centres will be conducted in accordance with Good Clinical Practices and ICH. Medical and administrative records and CRFs will be kept for the duration of the study in the service and then archived for a minimum of 30 years. In accordance with article R.5120 of the French Public Health Code, the investigators, as well as any persons collaborating in the study, will respect medical confidentiality especially as concerns the nature of the study, the persons participating in the study and the obtained results.

On all study-related documents, the patient will be identified using only a unique, 7-character identification number, and the first letter of his/her last name, the first letter of his/her first name and his/her year of birth. A patient identification list will be maintained by the investigator (and only the investigator). The investigator will ensure that the anonymity of each person involved in the study is respected. No identifying information will be disclosed to third parties other than those statutorily entitled to hold this information (and who are bound by professional secrecy).

### Plans for collection, laboratory evaluation and storage of biological specimens for genetic or molecular analysis in this trial/future use {33}

No biological data will be collected during this study.

### Statistical methods

#### Statistical methods for primary and secondary outcomes {20a}

The statistical analysis will be performed by the Department of Biostatistics, Epidemiology, Public Health, and Health Economics of Nîmes Hospital Center using SAS statistical software, Carey, NC. A difference will be considered as statistically significant if the test gives a *p* value of 0.05 or less.

##### Description of the population included and main parameters studied

Initial data analysis will describe the sample and the population per group. The Shapiro-Wilk test will be used to determine whether or not the quantitative variables show a normal distribution. Statistical results will be presented in the form of “mean ± standard deviation” for quantitative variables showing a normal distribution, and “median and interquartile intervals” for other variables. The number and associated percentage will be given for qualitative variables.

##### Analysis of the principal endpoints

The duration of a suicide reattempt-free follow-up period (SRFFP) will be assessed in the two groups using the Kaplan-Meier method and compared by a log-rank test. This analysis will be completed by a modeling analysis to take into account clustering effects.

Indeed, in our study, randomization to treatment is done on an individual basis. However, the experimental treatment is administered to a group so that several individuals receive the intervention together by the same therapist. Observations within the group therapy will likely be correlated within groups (clustering effect). In contrast, control participants receive an individual intervention and their observations can reasonably be assumed to be independent. Either arm may also be influenced by cluster effects linked to a particular therapist. The latter clustering results in asymmetric, partially nested designs. Recently, statistical models were developed to appropriately evaluate treatment effects when using a partially nested design [29]. Based on these, we will use multilevel mixed-effects models to assess the treatment effect on the outcomes describing the suicidality:

- The duration of a suicide reattempt-free follow-up period (SRFFP),
- The suicide reattempt during the follow-up (yes/no) by adjusting for nested effects,
- The C-SSRS score at 12 months by adjusting for nested effects and C-SSRS score at inclusion.

The detailed statistical plan will be provided before data extraction and unblinding.

The models will also provide valuable estimates of intracluster correlation coefficients for the different psychological outcomes of our study in the context of behavioural group therapy; these data are necessary to optimize the sample size of further studies in the area of psychological research.

##### Analysis of the secondary endpoints

Multilevel mixed-effects models will be used to assess treatment effects on the evolution of the C-SSRS score and the suicidal ideation (BSSI score) and of the psychiatric symptoms (BDI-II, BHS).

##### Guess-the-group

To control for the success of blinding, outcomes assessor responses to the “guess-the-group” question will be compared to true responses using the Kappa agreement coefficient.

##### Methods used to manage data that are missing, unused or invalid

For the primary analysis, the data are censored for the primary outcomes (SRFFP); it is not relevant to replace missing values. For the other outcomes, we do not have a replacement method for missing data.

##### Choice of patients to be included in the analyses

All patients included and randomized in the study will also be included in the analysis (intent-to-treat analysis). The conclusions of the study will be based on this analysis. Exploratory Per protocol analysis will be also performed.

#### Methods for any additional analyses (e.g., subgroup and adjusted analyses) {20b}

Additional analyses are not required.

#### Interim analyses {21b}

Interim analyses are not required.

#### Analysis to handle protocol non-adherence and any statistical methods to handle data {20c}

For the primary analysis, the data are censored for the primary outcome (SRFFP); it is not relevant to replace missing values. For the other outcomes, we do not have a replacement method for missing data.

#### Plans to give access to the full protocol, participant level-data and statistical code {31c}

Currently, the NUH does not support public access to trial documents.

### Oversight and monitoring

#### Composition of the coordinating centre and trial steering committee {5d}

Due to the primary outcome (suicide reattempt), a Data Monitoring Safety Committee will be formed and will include two psychiatrists and a methodologist. Members must be independent of the study and not employed by the study sponsor.

The monitoring committee is responsible for the following:

- Providing independent medical expertise when necessary
- Judging the severity of adverse events
- Preparing a notice of termination of the research in case of adverse events considered severe, not clearly established as being unrelated to the research, and which may jeopardize the health of patients

#### Composition of the data monitoring committee, its role and reporting structure {21a}

A sponsor-delegated clinical research assistant will regularly visit each of the study centers during the implementation of the trial. One or more visits will be carried out during the trial according to the rhythm of the inclusions and the duration of the study.

The reasons for these visits are:

- To verify that the protocol is being respected
- To verify the consent forms
- To verify serious adverse event reporting
- To carry out quality control: to compare case report form data with source document data within each centre

Those responsible for the quality control of this biomedical research trial and thereunto duly authorized by the sponsor have access to the individual data strictly necessary for quality control and are subject to professional secrecy.

All monitoring visits are accompanied by a written monitoring report (visit traceability).

#### Adverse event reporting and harms {22}

The events and adverse events defined for each type of research are reported respectively by the investigator to the sponsor and the sponsor to the competent authority and the appropriate committee for the protection of persons. In this case, the committee shall ensure, if necessary, that people involved in the research were informed of any potential adverse events/side effects and that they confirm their consent. The patients included in the trial will be monitored for 12 months. The monitoring of complications and adverse events is scheduled in line with Fig. 1. Any emergencies will be managed by the investigator. Any patients who experience an adverse event (or not) will be followed up by the physician until complete resolution of the complication. Following study completion or end, follow-up is continued as decided by the investigator.

When a new event happens in relation to a research trial or a product covered by a research trial and this event is likely to prejudice the safety of participating persons, the sponsor and the investigator shall take appropriate, urgent, safety measures. The sponsor shall promptly inform the competent authority and the committee for the protection of persons of these developments and, where appropriate, any actions that were taken.

#### Frequency and plans for auditing trial conduct {23}

Investigators agree to comply with the requirements of the sponsor and the Competent Authority in respect to audits or inspections of the study. An audit can cover all stages of the study, from protocol development to publication of results and the classification of the data used or produced as part of the study.

#### Plans for communicating important protocol amendments to relevant parties (e.g. trial participants, ethical committees) {25}

Any substantial change, that is to say, any changes that might have a significant impact on the protection of persons, the conditions of validity and the results of research, on the quality and safety of the interventions tested, on interpretation of scientific documents that support the conduct of research or the modality of conduct, will be the subject of a written amendment that is submitted by the sponsor to the Committee for the Protection of Persons (CPP) and the competent authority for approval prior to being implemented.

Insubstantial changes, that is to say those that have no significant impact on any aspect of research whatsoever, are transmitted to the CPP in order to inform the CPP of such changes.

All amendments to the Protocol must be brought to the attention of all investigators involved in the research. Investigators are obliged to respect their content.

Any amendment that modifies the care of patients or the benefits, risks and constraints of the research is the subject of a new briefing note and a new consent form which requires the same collecting procedures as mentioned above.

### Dissemination plans {31a}

The results of this study will be presented in a peer-reviewed scientific journal and in oral communication at scientific conferences. It is not expected to resort to medical writers. Authorship follows the guidelines set by the International Committee of Medical Journal Editors (ICMJE). Currently, the NUH does not support public access to trial documents.

## Discussion

This is the first randomized controlled trial to evaluate a manualized group cognitive behavioural therapy for preventing repeat suicide attempts. The expected results of this study are likely to have multiple benefits.

For the patient, demonstrating the efficacy of GPACTS to prevent repeat suicide attempts will provide relief from the mental suffering that occurs in the aftermath of a suicide attempt. Repeat suicide attempt prevention also constitutes in itself a direct and immediate therapeutic advantage. If proven effective, this therapy would greatly improve the management of suicidal patients.

Considering the economic burden represented by the management, particularly in terms of hospitalization, of patients following a suicide attempt, an important benefit is expected in terms of public health. If proven effective, GPACTS would provide a less-expensive option (when compared to individual therapy) and a pragmatic solution for the prevention of repeat suicide attempts.

### Innovative aspects

Here we describe the study protocol of an RCT comparing GPACTS versus IST that will provide sound results on which to base recommendations for the prevention of new suicide attempts among patients currently seeking treatment for SB. According to Tarrier et al. [7], the number of studies that compared CBT with another active treatment was comparatively low, and to our knowledge, no team has yet to compare GPACTS with IST for the prevention of repeat suicide. To our knowledge, this is the first RCT of its kind to be conducted in France and so far there are no studies in the literature on group psychotherapy for the treatment of individuals who have attempted suicide. In a randomized controlled trial, Brown et al. [6] demonstrated the effectiveness of a 10-session cognitive therapy to prevent repeat suicide attempts for adults who recently attempted suicide. Participants in the cognitive therapy intervention had individual face-to-face sessions. In our study, we will rather offer a group therapy intervention that should prove to be efficient while reducing the cost of care.

## Trial status

The current protocol version (5.0) was approved on 15 October 2018. The study is currently ongoing. Recruitment of patients started in November 2017 and will be completed in June 2020. The first groups, one that started with GCBT and the other with IST, received treatment from December 2017 to January 2018. The trial is currently ongoing.

## Data Availability

Raw data will be available from the corresponding author upon reasonable request. Transfer of clinical data will require approval from the Institutional Review Board.

## Abbreviations

BDI: Beck Depression Inventory
BESPIM: Department of Biostatistics, Epidemiology, Public Health and Medical Information at the Nîmes University Hospital
BHS: Beck Hopelessness Scale
BIS: Barratt Impulsiveness Scale
BSSI: Beck Scale for Suicide Ideation
C-SSRS: Columbia Suicide Severity Rating Scale
CBT: Cognitive behavioural therapy
CECA-Q: Childhood Experience of Care and Abuse Questionnaire
CépiDc: Center for Epidemiology on Medical Causes of Death register
CPP: Committee for the Protection of Persons
eCRF: Electronic Case Report Form
GPACTS: Group Post-Admission Cognitive Therapy for Suicidality
ICH: The International Conference on Harmonisation of Technical Requirements for Registration of Pharmaceuticals for Human Use
ICMJE: International Committee of Medical Journal Editors
IST: Individual Supportive Therapy
MINI: Mini International Neuropsychiatric Interview
MIT-IQ: Cognitive Reflection Test
NUH: Nîmes University Hospital
PACT: Post-Admission Cognitive Therapy
PSRFFP: Probability of a suicide reattempt-free follow-up period
RCT: Randomized controlled trial
RRRS: Risk-Rescue Rating Scale
SB: Suicidal behaviour
SPIRIT: Standard Protocol Items: Recommendations for Interventional Trials
SRRS: Social Readjustment Rating Scale
STAI: State-Trait Anxiety Inventory
STAXI: Spielberger’s State-Trait Anger Expression Inventory
